# Endograft rescue of compromised interposition aortic graft in an adult patient with congenital heart disease

**DOI:** 10.21542/gcsp.2018.8

**Published:** 2018-03-14

**Authors:** Jesse W Lee, Kanishka Ratnayaka, Howaida G El-Said, John W Moore

**Affiliations:** Department of Pediatrics, Division of Pediatric Cardiology, University of California, San Diego and Rady Children’s Hospital, San Diego CA, USA

## Abstract

In a 19-year-old male with interrupted aortic arch and complex congenital heart disease, we report percutaneous repair of a compromised aortic conduit. The patient had aortic arch repair in childhood utilizing a 12 mm Hemashield Dacron conduit. CT angiography showed multiple segments of this conduit were dilated to 16 mm suggesting conduit degeneration and failure with pseudoaneurysm formation. We utilized a self-expanding aortic endograft supported by internal placement of bare metal stents to repair the conduit. Our repair was guided by 3D rotational angiography. This adult patient with complex congenital heart disease and interrupted aortic arch is an example of patients in whom endograft repair of compromised aortic conduits presents a much lower risk alternative than surgical revision.

## Introduction

The prevalence of adult congenital heart disease is rising in the United States. As a result of advancements in their care, adults (over 18 years of age) now outnumber children with congenital heart disease. Among these adults, there are a significant number with early repairs that have become compromised^1^. Surgical reoperation in these patients may be technically difficult and have associated significant risk. New percutaneous strategies with lower risk are needed, and one new strategy for a group of patients is illustrated by this case. 

## Case report

**Figure 1.**
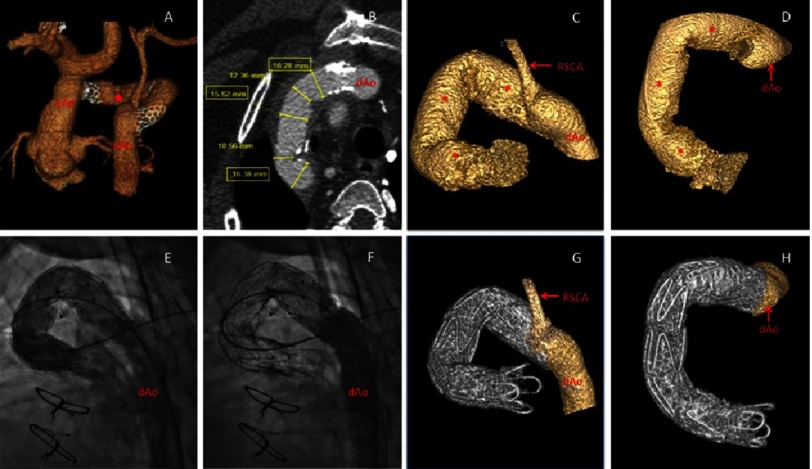
A 19-year old with complex adult congenital heart disease including interrupted aortic arch type B2 post repair with interposition graft now with dilated segments concerning for loss of structural integrity, stenosis, and folding. A, CT angiogram 3D reconstruction shows repaired aortic arch with interposition graft (⧫). Previously placed proximal and distal stents are visible with stenosis. B, CT axial image of the interposition graft showing stenosis and ominous dilated segments. C–D, Pre-intervention 3D rotational angiography (3DRA) was used to delineate complex anatomy, determine optimal imaging angles, and plan endovascular approach. Areas of dilation (*). E, Fluoroscopic contrast angiogram post endovascular graft (Cook Medical, Bloomington, IN) deployment in optimal working angle derived from 3DRA. F, Repeat angiogram post bare metal stenting within the endovascular graft. G–H, Post-intervention 3DRA shows endovascular repair of previously compromised interposition graft.

A 19-year-old male having complex congenital heart disease (dextrocardia, situs inversus, double outlet right ventricle with L-malposition of the great arteries, and interrupted aortic arch with an aberrant right subclavian artery, type B2) had complete repair in infancy using a very small aortic conduit. The patient had revision of the repair utilizing a 12 mm Hemashield Dracron conduit at 23 months of age. At 19 years, he was asymptomatic if sedentary; however he developed shortness of breath and chest pain with minimal exercise (NYHA Class II). Computerized tomography (CT) angiography showed compromised graft integrity with multiple segments dilated to 16 mm suggesting pseudoanurysm formation. In addition, there were areas of stenosis and kinking along the conduit’s course (Figure 1A–B). Surgical options were complex and carried significant risk^2^. Joint surgical and interventional consultation recommended pursuit of percutaneous therapy.

After informed consent, the patient was brought to the cardiac catheterization laboratory, intubated, and placed under general anesthesia. Left femoral vessels were accessed percutaneously in a standard fashion using ultrasound guidance. Left and right heart diagnostic catheterizations were performed. Hemodynamics showed a 53 mmHg systolic gradient across the interposition graft. Three-dimensional rotational angiography (3-DRA) further delineated multiple areas of dilation, stenosis, and kinking of the aortic graft (Figure 1C–D, supplemental video). 3-DRA was used to determine optimal imaging angles and plan placement of an endovascular graft within the in situ compromised interposition graft for reinforcement (Figure 1E). A Lunderquist 0.035” guidewire (Cook Medical, Bloomington, IN) was positioned into the left ventricle. The aortic sheath was upsized to a 16 Fr sheath. A 18 mm x 105 cm Zenith Alpha™ Thoracic Endovascular Graft (Cook Medical, Bloomington, IN) was prepared and advanced across the conduit aortic interposition graft.

After confirming position, the self-expanding endovascular graft was deployed by retracting the delivery sheath. Due to a significant residual gradient after the graft was deployed, four balloon expandable bare metal stents, Palmaz Genesis 2910B (Cordis, Milpitas, CA), Max LD 36 mm (Covidien, Plymouth, MN), were subsequently implanted within the graft to correct stenosis from conduit narrowing and kinking (Figure 1F). These stents were dilated to 18 mm making the diameter a full 18 mm along the entire course of the conduit. Post-intervention 3-DRA demonstrated endograft covering of the entire length of interposition graft, including concerning dilated segments, as well as relief of stenotic and kinked portions of the conduit (Figure 1G–H, supplemental video). Aberrant right subclavian artery patency was preserved. After this procedure, the systolic pressure gradient across the graft was reduced to 9 mmHg. The patient recovered uneventfully and was discharged the day following the procedure. The patient was treated with daily low dose aspirin.

Follow-up of the patient to one-year post procedure suggested no residual clinical problems and improved exercise tolerance (NYHA Class I). Follow-up CT angiogram showed no distortion of the conduit stents and echocardiography suggested no significant conduit gradient.

## Discussion

Complex adult congenital heart disease patients present new management challenges. Dacron conduits, previously used in repairs of pediatric patients, may lose integrity over time leading to pseudoaneurysms and rupture when the patients are adults^3,4^. Surgical revisions in such adult patients are undesirable due to a high risk of uncontrollable bleeding.

In this case, the interposition graft had areas of clinically significant stenosis complicated by pseudoaneurysm formation suggested by the areas of dilation. Percutaneous therapy with bare metal stents alone would risk uncontained rupture of the compromised conduit. A large diameter aortic endograft, designed for degenerative aortic disease, was used in this adult patient to repair the surgical interposition graft placed at two years of age. It allowed for bare metal stenting to enlarge the conduit to appropriate size with minimal risk of bleeding and rupture. 3-DRA was useful in this case as it aided in delineating the complex structure of the failed conduit, in determining optimal two dimensional fluoroscopic imaging angles during the procedure, and in positioning the endograft and the bare metal stents.

## Conclusion

This report describes the use of a percutaneously placed endovascular graft to rescue a compromised interposition aortic graft in an adult congenital heart diseased patient where surgical options were complex and carry significant risk for mortality and morbidity. We highlight the importance of multimodality imaging in complex congenital heart disease and the potential applications for currently available endovascular grafts in a growing population.

## References

[1] Gilboa S, Devine O, Kucik J, Oster M, Riehle-Colarusso T, Nembhard W, Xu P, Correa A, Jenkins K, Marelli A (2016). Congenital heart defects in the United States: estimating the magnitude of the affected population in 2010. Circulation.

[2] Marcheix B, Lamarche Y, Perrault P, Cartier R, Bouchard D, Carrier M, Perrault LP, Demers P (2007). Endovascular management of pseudo-aneurysms after previous surgical repair of congenital aortic coarctation. European Journal of Cardio-Thoracic Surgery.

[3] Riepe G, Loos J, Imig H, Schröder A, Schneider E, Petermann J, Rogge A, Ludwig M, Schenke A, Nassutt R, Chakfe N, Morlock M (1997). Long-term in vivo alterations of polyester vascular grafts in humans. Eur J Vasc Endovasc Surg.

[4] Wilson S, Krug R, Mueller G, Wilson L (1997). Late disruption of Dacron aortic grafts. Ann Vasc Surg.

